# A commentary on ‘Clinical results of a 10-year follow-up of surgical treatment for elbow stiffness in rheumatoid arthritis: A case series’

**DOI:** 10.1097/JS9.0000000000001620

**Published:** 2024-05-09

**Authors:** Ying Zhu, Chujun Luo, Xinchang Wang

**Affiliations:** a The Second Clinical Medical College of Zhejiang Chinese Medical University; b The Second Affiliated Hospital of Zhejiang Chinese Medical University, Hangzhou, Zhejiang, People’s Republic of China

*Dear Editor,*

We are interested in the article entitled ‘Clinical results of a 10-year follow-up of surgical treatment for elbow stiffness in rheumatoid arthritis: A case series,’ which was published in the *International Journal of Surgery* ^1^. Rheumatoid arthritis (RA) is a chronic inflammatory autoimmune disease, one of the major disabling diseases in the world^2^. RA is characterized by chronic inflammation of the synovial membrane of the joints, with synovial hyperplasia and infiltration of a variety of inflammatory cells, which ultimately leads to the destruction of cartilage and bone, resulting in limited joint activity and limb disability^3^. Disease activity and bone erosion have always been clinical concerns, and controlling RA synovial inflammation, decreasing disease activity, and delaying bone destruction are the primary issues for clinical research. This study evaluated the long-term outcomes of surgical treatment in a consecutive series of rheumatoid arthritis patients with elbow stiffness and early joint destruction. This is a retrospective study with a specialized assessment of the status at 10 years postoperatively. This study concluded that surgical treatment is a reliable approach to rheumatoid elbow stiffness, effectively improving elbow mobility, function, muscle strength, pain relief and alleviating neurologic symptoms. Although this study has some clinical significance, there are still several issues that deserve to be explored in depth.

Firstly, it is commendable that this study used a retrospective study to systematically review patients with rheumatoid arthritis with elbow stiffness who underwent surgical treatment over a 10-year period and that multiple patient-specific data were collected independently by three senior surgeons to control for bias. However, only 43 patients ultimately met the criteria after cascade screening, a relatively small sample size that resulted in low statistical power of the results. The inclusion of eligible patients can be continued in future studies, and increasing the sample size will enhance the robustness and reliability of the results.

Secondly, this study adopted a single-center study design, which may create some bias, and future multicenter studies could be conducted, which would both expand the sample size and facilitate communication between researchers in different regions. Multicenter studies have valuable benefits for all clinical researchers. However, it is worth noting that variations in surgical procedures, the level of experience of the surgeon, and the patient’s physical fitness may have a considerable impact on the patient’s recovery and, thus on the results of the study.

Thirdly, the study’s treatment of potential confounders is commendable as it effectively adjusted for several key covariates including sex, age, side of disease, body mass index (BMI), smoking status, diabetes, and hypertension. Clinical characteristics such as duration of follow-up, duration of rheumatoid disease, duration of elbow stiffness, elbow function, muscle strength, neurologic symptoms, complications, and recurrence were also recorded. However, the robustness of the findings may be further enhanced by taking into account many other potential confounding factors that may affect the results, such as the patient’s own fitness, the surgeon’s level of experience, and the patient’s adherence to postoperative rehabilitation, postoperative analgesia, and antirheumatic therapy.

In addition, the 10-year follow-up period of this study may reflect to some extent the improvement of the quality of life of the patients by the surgery, but the study was a single-center, small-sample study, and the postoperative recurrence rate and probability of complications were not representative of the overall population and could not be analyzed statistically enough to provide a comprehensive assessment of the disease. Extending the duration of follow-up may have yielded more reliable results. Moreover, if patients are not on regular antirheumatoid therapy, it may cause a recurrence of synovitis, and patients may be monitored for synovitis in subsequent studies.

Overall, this study treated many patients with elbow stiffness due to untreated rheumatoid arthritis or failure of conservative treatment, and the patients were found to have a high quality of life after observational follow-up. Existing rheumatoid arthritis patients are often seen in rheumatology departments, where physicians rely on medications to relieve the pain of rheumatoid arthritis. However, many patients with rheumatoid arthritis often develop joint deformities in the late stages of the disease, which severely affect their quality of life and cannot be relieved by medication alone. Surgery is an effective complementary approach to failed or untimely conservative treatment of RA, and standard rehabilitation improves elbow mobility, muscle strength, and function. This study uses surgical methods to address some of the pain in these patients and may provide a surgical solution for the treatment of rheumatologic diseases, where combined medical and surgical treatments will maximize the quality of life of patients. We look forward to more groundbreaking contributions from the authors in their future work.

## Ethics approval

Not applicable.

## Consent

Not applicable.

## Sources of funding

This work was supported by a grant from the National Natural Science Foundation of China (No. 82374402).

## Author contribution

Y.Z., C.L., and X.W.: conception and design of the study; Y.Z. and C.L.: collected and analyzed the data; Y.Z. and C.L.: write the paper. All the authors drafted and revised the paper. In addition, all authors read and approved the final manuscript.

## Conflicts of interest disclosure

The author declares no conflict of interest.

## Research registration unique identifying number (UIN)

Not applicable.

## Guarantor

Xinchang Wang accepts full responsibility for the work and/or the conduct of the study.

## Data availability statement

This manuscript is a comment. Don’t need a data availability statement. However, all the data from the current study are publicly available.

## Provenance and peer review

Not applicable.
